# Self-control has a social role in primates, but not in other mammals or birds

**DOI:** 10.1038/s41598-025-99523-6

**Published:** 2025-05-21

**Authors:** R. I. M. Dunbar, Susanne Shultz

**Affiliations:** 1 Department of Experimental Psychology, Anna Watts Building, University of Oxford, Oxford, OX2 1GG UK; 2 Department of Earth and Environmental Sciences, Michael Smith Building, University of Manchester, Manchester, M13 9PT UK

**Keywords:** Primates, Bonded social groups, Self-control, Inhibition, Causal reasoning, Temporal discounting, Ecology, Behavioural ecology

## Abstract

The capacity to inhibit prepotent actions (strategic self-control) is thought to play an important role in many aspects of the behaviour of birds and mammals. Though widely considered important for foraging decisions, inhibition is also crucial for maintaining the temporal and spatial coherence of bonded social groups. We analyse four different sets of comparative experimental data on primates to show (1) that tasks widely assumed to index inhibition segregate naturally into two distinct clusters (those that involve strategic self-control and those that might be better described as detour or causal reasoning tasks) and (2) that, across primate species, the former tasks correlate better with the demands of social contexts, while the latter correlate better with the demands of foraging contexts. Finally, using a wider sample of mammals and birds, we show (3) that the capacity for (strategic) self-control is unique to anthropoid primates (as suggested by the Passingham-Wise Conjecture). We propose that strategic inhibition may be neurologically costly (and hence taxonomically rare) because animals have to model two different views of the world at the same time and prevent one leaking into the other. We conclude, first, that future studies need to examine the cognitive demands of the tasks they use more carefully and avoid misusing terms to label phenomena that involve very different demand characteristics and, second, that more attention is given to neuroimaging studies that examine the neural activity involved in different tasks.

**Supplementary Information:**

The online version contains supplementary material available at 10.1038/s41598-025-99523-6.

## Introduction

The capacity to inhibit prepotent responses (self-control) forms part of the suite of advanced cognitive skills known as executive functions that play an important role in the behaviour of animals and humans^1,2^. The concept of inhibition itself has, however, generated debate in at least three different respects: its cognitive basis, its functional domain(s) and its taxonomic distribution. Conceptually, it is helpful to distinguish between tactical forms of motor (or action) inhibition (the capacity to inhibit a behavioural response, as in switching from one learned pattern or habit to another following a change in reward schedules, or choosing between two alternative simultaneous rewards) and strategic inhibition (suppressing a prepotent action in the hopes of obtaining a better option at some future time, as in temporal discounting or delay of gratification tasks)^1,2^. These are not the same: in the first, the reward is in full view whereas, in the second, the choice is between a reward that is physically present and a future reward that can only be imagined. At the functional level, the issue is whether these capacities are general cognitive abilities that underpin a wide range of behavioural functions or specialist ones likely to have evolved in response to a specific selection pressure. In taxonomic terms, the issue is whether these traits are widespread or restricted to specific taxonomic groups.

Many studies have suggested that self-control plays a central role in foraging^3–6^ on the grounds that, in order to forage optimally, animals have to be able to forego a less valuable immediate reward in order to gain a more desirable future one^3^. However, inhibition can also be important in the social domain. This possibility has largely been overlooked because of an emphasis in experimental studies on immediate rewards driven largely by the need for simple experimental designs that produce rapid, measurable outcomes. Social outcomes, in contrast, are much less convenient and much less easy to measure (for example, many can only be measured at the end of a lifetime). In species that live in stable social groups (as opposed to herds and flocks), for example, the capacity to maintain the social cohesion of a group has very significant consequences for the fitness of its members, but measuring that at the individual level is difficult, not least because social cohesion is the property of a set of animals over time not of an individual animal at a particular time point.

That social variables may be cognitively demanding can be illustrated by comparing herd-living species like ungulates that live in unstable groups (aggregations) with species like anthropoid primates that live in permanent, demographically stable groups (congregations). In herding species, animals readily drift apart during the course of the day’s foraging because their activity schedules and speed of movement get out of synchrony^4–12^; their social interactions and spatial coordination are based mainly on anonymous interactions and simple behaviour matching (as in the aerial displays of some bird flocks^13,14^). In contrast, bonded social groups remain cohesive and spatially coordinated during foraging because individuals are willing to suspend (i.e. inhibit) foraging when others want to rest, or cease resting when others want to forage in order to avoid becoming separated^15–22^.

Equally important may be the capacity to hold back from attacking others unnecessarily: too much aggression may cause a group to fission, thereby reducing its size and hence the benefits it provides. Similarly, vicarious attacks on individuals who have allies willing to come to their aid are not only socially disruptive but also risk injury to the aggressor^23^. In a seminal series of field experiments on wild hamadryas baboons, Kummer et al.^24^ showed that males inhibit their willingness to challenge a rival male for his female if the female is exhibiting close interest in her male – even when the male knows from prior experience that he can defeat the rival in dyadic contests (a phenomenon referred to as “triadic differentiation”). Similar behaviour has been noted in gelada baboons^25^. In humans, the ability to delay gratification in childhood strongly predicts future social skills as an adult, with poor capacity to do so being directly related to disruptive anti-social behaviour, getting into trouble with the law and an inability to maintain stable relationships^26–29^, all of which are indicative of an inability to negotiate compromises. All these responses are highly sensitive to context and would seem to involve high order cognitive processing beyond the level of simple responses to changing reward schedules. Such behaviour often requires animals to understand third party relationships (widely documented among cercopithecine monkeys and apes, but not other species^30^), as well as being able to selectively inhibit actions likely to have adverse future consequences.

Both the difficulty of maintaining group cohesion in species that form stable social groups and the cognitive demands of doing so have been gravely underestimated by the kinds of experimental designs widely used to study cognition^30,31^. Individual differences in the social skills that allow animals to create and maintain bonded^32^ relationships are, for example, highly correlated with a variety of fitness indices in a number of taxa. Individuals who have more social partners experience lower stress levels, suffer less from illnesses, recover faster from injury, live longer, have higher fertility, and have offspring that are more likely to survive and reproduce in their turn than socially less well embedded individuals (chimpanzees^33^, baboons^34–39^, macaques^40–44^, equids^45^, hyaena^46^, dolphins^47,48^). These results concur with a very substantial human literature showing that socially well embedded individuals experience similar health benefits^49–51^. Yet these outcome indices are never considered in experimental designs – in part, perhaps, because it is not possible to force animals to have meaningful social relationships through experimental manipulations and because the outcomes often require years to measure.

What characterises these examples is a trade off between short and long term benefits (i.e. between immediate and future rewards). Being able to evaluate the consequences of one’s behaviour on a long time horizon is cognitively demanding compared to simple motor inhibition because it entails being able to imagine a virtual world in which alternative possibilities can be compared. Neuroimaging studies suggest that the cognitive processing of mental states requires the recruitment of considerable additional neural input over and above that required for making judgments about the simple physical aspects of behaviour^52^.

We can expect the simpler forms of behavioural inhibition to be taxonomically widespread because it forms a core part of association learning. Indeed, there is considerable experimental evidence that birds and small-brained mammals like rodents are readily able to learn reversal tasks that require them to switch between responses when reward schedules change^53–56^. In contrast, the kinds of behaviours associated with maintaining stable social groups (strategic inhibition) seem to be restricted to large-brained Old World monkeys and apes^30,56^. Despite this, the taxonomic distribution of self-control remains contested, with some arguing that it is limited to anthropoid primates^57^, others that these skills are widespread in advanced vertebrates generally (mammals^58–61^ and birds^62–65^), while others would include even fishes^66^. Conflicting claims of this kind often result from using tasks that assay different cognitive abilities^67–69^, or are a consequence of falling foul of definitional slippage associated with the ‘sloppy proxy’ syndrome^70^.

Neuroimaging experiments and lesion studies can provide helpful insights in this respect. There is comparative evidence, for example, to suggest that inhibition competencies (broadly defined) correlate with brain size in primates, but may not do so in other mammals and birds^58,59^. Finer scale studies suggest that, in both humans and rodents, simple motor inhibition (e.g. Go/No-Go tasks) and foraging decisions typically activate the medial PFC (including the ACC [anterior cingulate cortex: Brodman Areas BA24/32/33], a brain region associated with error detection and violations of expectation)^53,71,72^, whereas strategic inhibition (self-control) involves the frontal pole (BA10^57^) and the ventrolateral prefrontal cortex (BA44/45^73^). Passingham & Wise^57^ proposed, on the basis of extensive behavioural and lesion studies on rodents, marmosets, macaques and humans, that self-control depends on a brain region, the frontal pole (BA10), that is only found in anthropoid primates (the Passingham-Wise Conjecture)^57^. Boschin et al.^74^ combined a battery of seven reasoning tasks with lesions in monkeys to show that BA10, in particular, is crucial for rapid learning about the relative value of alternative actions. Between them, these observations provide additional grounds for questioning whether feeding decisions involve high order self-control, and hence that this cognitive ability might have evolved for other purposes.

We address these issues by reanalysing published experimental data on a variety of commonly used inhibition tasks in order to determine (1) whether they all index the same underlying cognitive ability, (2) whether or not they have the same behavioural function and (3) how widely distributed they are taxonomically. To test the first, we use data from two primate datasets to determine how closely different tasks agree with each other. To test the second, we use data from four different primate datasets to test whether these tasks correlate best with indices of social or ecological decision-making. Finally, we use a dataset that tested two tasks on a large sample of mammal and bird species to formally test the Passingham-Wise Conjecture.

## Methods

### Data

For our primary analyses, we use data from three independent studies that ran the same experimental protocols on between 7 and 18 primate species (Table 1). Amici et al.^75,76^ and MacLean et al.^58^ carried out a series of experiments on different species using the same experimental protocol, with all the animals doing all the tasks in most cases; in contrast, Stevens^59^ collated data from the literature where different studies had used a temporal discounting indifference task to determine the time delay at which animals are no longer willing to delay for a larger reward on a variety of primate species. The experimental protocols are the same in the MacLean and Amici A-not-B task and in the Stevens and Amici temporal discounting task. The tasks and their characteristics are listed in Table 2. In addition, we use data on nine standard executive function tasks collated from the literature by Shultz & Dunbar^77^ for a set of 39 primate species (representing 21 genera). In this case, the species were sampled unequally on an average of 2.7 tasks (range 1–8) per species. The tasks and species sampled are given in Table S1. Table S2 indicates the range of scores across the species sampled, and Fig. S1 indicates the relative difficulty of these tasks (as indexed by the mean percentage of correct trials by a range of primate taxa).

**Table 1:** Primate species sampled and sample size (number of individuals sampled).

	MacLean et al.^58^	Stevens^59^	Amici et al.^75^	Amici et al.^76^
Eulemur fulvus	10			
Eulemur macacao	14	1		
Eulemur rubiventer	8			
Lemur catta	15			
Propithecus cockereli	16			
Varecia rubra		1		
Varecia variegata	18	3		
Saguinus oepidus		6		
Callithrix jacchus	12	5		
Leontopithecus rosalia	10			
Sapajus apella	36	12	27	8
Ateles geoffroyi	13	12	16	6
Saimiri sciureus	19			
Rhinopihecus roxellana	10			
Macaca mulatta	6			
Macaca fascicularis	12	14	12	
Macaca arctoides	7			
Papio anubis	9			
Papio hamadryas	12			
Pongo pygmaeus	17	8	10	5
Gorilla gorilla	15	4	7	4
Pan paniscus	7	5	4	5
Pan troglodytes	17	5	8	6

**Table 2:** Tasks used in the various studies.

Study	Task	Description
Stevens^59^	indifference	time delay at which animals is no longer willing to delay for a larger reward
MacLean et al.^58^	A-not-B	select new position when rewarded cup is moved after baiting in a 3-cup task
	cylinder	select ends of transparent cylinder not middle of tube to access reward
Amici et al.^75^	A-not-B	as above
	middle cup	spatial displacement: ignore middle cup when reward is moved in a 3-cup task
	plexiglass hole	reach through side hole rather than through plexiglass screen when reward is moved from in front of one of 2 holes to a position in between
	swing door	select opposite of 2 swing doors in plexiglass screen to avoid other door knocking reward behind it off shelf
	indifference	as above [labelled ‘delayed gratification’ in^75^
Amici et al.^76^	social task [ExpSR]	select smaller reward to avoid larger reward going to rival

We sourced mean group size for species from^78^, percentage of fruit in the diet (except for *Macaca mulatta*: see Table S3) from^79^, and day journey length (in km) and home range size (in ha) from^80,81^ and primary sources therein (for exceptions, see *ESM*). We exclude the domestic dog and the aye aye (*Daubentonia*) from the MacLean et al. dataset: the first because it is a highly inbred domestic species, and the latter because of uncertainties about its correct group size^56,78^. For detailed discussion on these exclusions, and on group size for *Pongo*, see *ESM.*

To test the Passingham-Wise Conjecture^57^, we use the data given by^58^ on the A-not-B and cylinder tasks for 36 mammalian and avian species. In this case, we ask simply whether major taxonomic groups differ in their performance on the two tasks.

The data are given in *ESM DATASET-1_inhibition tasks*,* DATASET-2_executive function tasks* and *DATASET-3_Passiungham-Wise Conjecture*, respectively.

### Statistical analysis

We first use maximum likelihood factor analysis on the Amici et al.^75^ dataset to determine whether their five tasks (all referred to as inhibition tasks) index the same capacity (i.e. are the product of the same latent variable). If they do, then the five tasks should form a single factor (i.e. performance on all five tasks will correlate highly). Conversely, if they form two or more distinct factors, this would imply that they index different underlying cognitive abilities.

We then use principal components analysis (PCA) with each of the MacLean^58^, Stevens^59^ and Amici^75^ datasets (adding the additional task from the later Amici et al.^76^ study in a follow-up analysis), as well as the Shultz & Dunbar^77^ executive functions dataset (though not all these tasks are inhibition tasks: see Table S1), to test whether the various inhibition tasks cluster better with a set of behavioural variables that reflect either the demands of maintaining group coherence during foraging or the demands of foraging decisions. We then use mediation analysis to determine whether these cognitive abilities mediate between brain size and either social group size or diet as representative of the two alternative outcome modes.

Finally, we use the full MacLean^58^ dataset (which includes data for a wide range of bird and mammal species in addition to primates) to test the Passingham-Wise Conjecture that only anthropoid primates have the capacity to inhibit prepotent responses. MacLean et al.^58^ do not compare performance against a null hypothesis of random choice. To provide a benchmark against which to compare correct decisions versus perseveration, we use H_0_ = 33.3% and 50% in A-not-B tasks (depending on whether we want to include the neutral “middle” cup), and H_0_ = 50% in the cylinder task (comparing entry attempts via the open ends versus entry attempts through the body of the tube: correct versus incorrect).

Although multiple regression has been widely used for testing hypotheses of this kind, the format of the standard regression model would oblige us to regress the cognitive cause (inhibition skill) onto the four ecological and social outcome variables, just as MacLean et al.^58^ and Stevens^59^ in fact did. Doing so, however, unavoidably reverses the natural causality (i.e. by assuming that behaviour determines cognitive ability rather than that cognitive capacity determines, or constrains, behaviour). Because this asks a very different question, doing so can yield seriously misleading results^70^. A statistically more elegant, if unconventional, approach is to use principal components analysis (PCA) to ascertain which variables covary (i.e. cluster together as a functional cluster). PCA avoids the need to presumptively specify the causal relationships between variables. We then supplement this with mediation analysis^82^ to test for causality in the relationship between the traits identified by the PCA.

As indices of foraging demand, we use the percentage of fruit in the diet and the size of the home range (or territory), both of which have frequently been used to test foraging demand hypotheses in comparative studies^58,59,79,83,84^. Fruits are much less predictable than foliage, and are usually viewed as representing a more cognitively challenging diet than leaves^83^. Similarly, large home ranges are assumed to be cognitively demanding both in terms of the mental mapping skills required to devise an optimal pathway between food sites^83^ and in the fact that foraging animals have to choose between near and distant locations on the basis of likely nutrient value^58,59^. In primates, both percent fruit in the diet and range size are strongly influenced by habitat conditions and hence impact directly on nutrient acquisition^85–89^. If self-control relates to foraging efficiency, performance on such tasks should correlate positively with these ecological indices: the more patchy and dispersed their typical food sources, the more the animals will need to be able to inhibit the temptation to stay in the current resource patch in order to take advantage of a richer food patch that is further away.

**Fig. 1:**
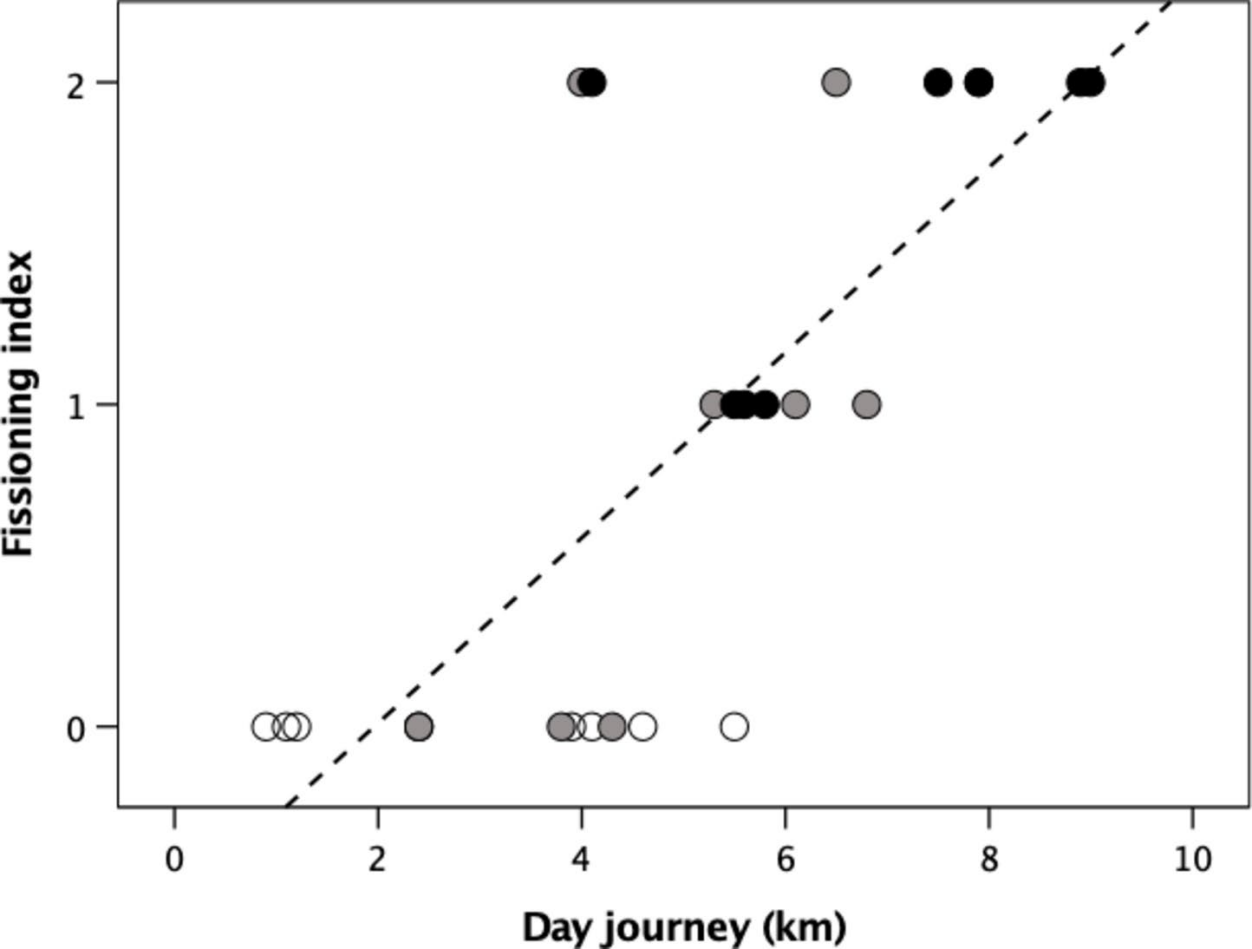
Fissioning index for individual *Papio* baboon populations as a function of day journey length. Unfilled symbols: group size < 35; grey symbols: group size 35–75; filled symbols: group size > 75. The data are given in Table S4. For definition of fissioning index, see Table S5.

In respect of the social domain, we focus on the role that self-control might play in ensuring group cohesion during foraging. For social species, coordination problems increase as a function of both group size and the distance animals have to travel, since both make it more likely that individuals’ activity cycles will progressively drift out of synchrony and lead to group fission^15,21,30^. Baboons provide a well studied example: the risk of group fragmentation increases as day journey lengths get longer and group sizes get larger (Fig. 1; Tables S3-S4). Our fissioning index correlates significantly with both group size (Kendall’s τ = 0.642, *N* = 26, *p* < 0.001) and day journey length (τ = 0.655, *N* = 26, *p* < 0.001). To determine whether there is an interaction effect between the two independent variables, we transformed group size and day journey length to standard normal deviates, and ran a multiple regression with an interaction effect. The results are given in Table 3. There are significant main effects, of approximately equal weight, but no interaction effect. If self-control is primarily a social skill that influences group cohesion, it should correlate positively with these two indices. We would not expect it to correlate with indices of foraging.

**Table 3:** Regression analysis of fission index for 25 different *Papio* study sites.

Variable	Slope	standardised β	df	t	p
Overall model: r^2^ = 0.664, F_3,21_=14.47, *p* < 0.0001.					
SD(group size)	0.481	0.546	22	2.20	0.039
SD(day journey)	0.373	0.423	22	2.17	0.041
Interaction	-0.182	-0.207	22	-1.07	0.295

It is important to be clear about the difference between range size and day journey length since, viewed superficially, both look like foraging-relevant variables. In fact, functionally they are very different, and especially so for primates. Unlike herding species, most primates are frugivores: they do not forage semi-randomly in their environment in the way grazers do, but rather move from one discrete resource patch to another, often at some considerable distance^90,91^. Range size determines the number of patches available to a group, but does not, of itself, determine either the number of patches visited each day or the length of the day journey (both of which are determined by group size^87^). Day journey length, by contrast, is largely a consequence of the size of the group and typical patch size, which between them determine the number of patches the group has to visit each day to satisfy its collective nutritional requirements. In other words, range size defines the distribution of food sources that animals can choose between (and hence the choices they make on where to forage), whereas day journey length is the means for visiting the required number of patches each day (but does *not*, of itself, determine which patches to visit). The first is a resource choice issue, the second a routing issue. Only the second has significant implications for maintaining group cohesion.

Note that we do not correct for phylogeny in these analyses. Previous analyses of both the behavioural and ecological variables^92,93^ and the cognitive variables^58,59,77^ have found no effect of phylogeny on any results. In primates, the phylogenetic signals for group size, percent fruit in diet, home range size and day journey length are low or close to zero^94^. This reflects the fact that primates are behaviourally extremely flexible^95^, and in consequence most of the variance in their behaviour (including group size) is environmentally, and not genetically, determined^87,95^.

All analyses were undertaken in SPSS v.29.

## Results

### Do different ‘inhibition’ tasks index the same underlying cognition?

We first use maximum likelihood factor analysis to determine whether Amici et al.’s^75^ five cognitive tasks (all tested on the same set of species) index the same underlying variable. Table 4 gives the results. It is clear that the five tasks partition into two subsets. One subset includes the A-not-B task (the same task as used by MacLean et al.^58^ and a delayed gratification task (a similar design to the temporal discounting task used by Stevens^59^). The other subset includes their Middle Cup, Plexiglass Barrier and Swing Door tasks. Although these latter tasks are presented as indexing inhibition (and were so presented by the sources from which the designs were taken), realistically they are in fact detour tasks^69^ and best understood as tests of (physical) causal understanding. Understanding that pushing a door will knock the reward off the shelf behind it whereas reaching through the adjacent door will allow the animal to reach the reward, for example, has much more to do with understanding material causality than being a matter of self-control.

**Table 4:** Factor loadings (maximum likelihood with varimax rotation) for the five cognitive tasks in the Amici et al.^75^ dataset.

Tasks	Factors	
	1	2
A-not-B	**0.805**	0.000
Middle cup	0.238	**0.641**
Plexiglass hole	0.394	**0.692**
Swing door	-0.135	**0.682**
Indifference	**0.912**	0.274
Species sampled	7	
Variance explained	62.8%	

Extraction based on λ = 1.0. Bold font indicates variables that have a strong positive load on the same factor.

Since the tasks within each subset weight approximately equally, we averaged the values to give a single value for each subset, referring to the first subset as inhibition tasks and to the second subset as detour tasks. We compared these two indices with the same species’ performances on the social inhibition task of Amici et al.^75^ and the MacLean et al.^58^ A-not-B and cylinder tasks. None of these tasks correlate significantly with each other, and several correlate negatively (Table 5; bivariate correlations for individual tasks are given in Table S6). Thus, despite having been presented in many publications as indices of inhibition, it seems that they in fact probably tap into very different cognitive skills. We therefore treat them separately.

**Table 5:** Bivariate Pearson correlations between the cognitive tasks provided by Amici et al.^75,76^ and MacLean et al.^58^.

Tasks	Amici et al.^75^	Amici et al.^76^	MacLean et al.^58^	
	detour task	Social inhibition	A-not-B	Cylinder
Amici inhibition (mean)	0.454 *p* = 0.153†	0.577 *p* = 0.115‡	0.385 *p* = 0.197†	0.661 *p* = 0.165*
Amici detour (mean)		**0.833** p = **0.020‡**	0.581 *p* = 0.086†	− 0.560 *p* = 0.780†
Amici social inhibition			0.110 *p* = 0.418‡	− 0.028 *p* = 0.986*
McLean A-not-B				-0.744 *p* = 0.628*

Notes:

(1) All p-values are the probability of a positive (1-tailed) correlation; bold values are significant.

† *N* = 7; ‡ *N* = 6; * *N* = 4.

(2) pairwise correlations for individual tasks are given in Table S5.

### Functional associations of the tasks

To determine whether the different cognitive variables associate with the four behavioural variables, we ran separate PCAs for the individual tasks. (Bivariate correlations between the Stevens and MacLean cognitive tasks and the four ecological outcome measures for the two larger datasets are given in Table S7 and Fig. S1.) For the Amici et al. (2008) dataset, we included both composite cognitive indices in the same analysis; the analysis for the inhibition task on its own is given in Table S8. With eigenvalues set to λ > 1, two factors are extracted for all five datasets, explaining > 70% of the variance in each case (Table 6). The three self-control (inhibition) tasks, group size and day journey length consistently load together on the same factor with high loadings, while diet is placed either in a separate factor or with the cylinder task. When the A-not-B and cylinder tasks are both included in the same analysis, the cylinder task loads more strongly with diet. Similarly, when the Amici et al. inhibition and detour tasks are included in the same analysis, a three factor model emerges (explaining 93% of the variance): home range and causal reasoning ability (cylinder task) are placed together in a separate factor from inhibition and the social indices, while diet loads on its own. In general, however, range size typically loads weakly across factors.

In case our choice of group size for *Pongo* distorted the results, we re-ran the three PCAs excluding this genus. Table S9a indicates that the results do not change (other than moving range area into the social factor and leaving diet isolated on the ecological factor). Table S9b confirms that including *Papio hamadryas* in the MacLean et al. analysis with alternative grouping sizes does not change the main results. Notice that, although the overall fit is slightly lower, the loadings for the smaller group size (considered their natural group size^56^) are a very close match to those in Table 6. *P. hamadryas* was not sampled in the other two datasets, so this species could not have biased the results in either of these cases. Table S10 confirms that these results hold when the data are analysed as genus level means, indicating little or no influence of phylogenetic autocorrelation. In short, potential confounds have not distorted the results.

For six of the species tested by^75^, Amici et al.^76^ provide data on a ‘social inhibition’ task (a form of reverse reward task: will animals resist reaching for the larger reward if doing so results in the rival getting it while they end up receiving the smaller reward? ). We ran a PCA for this index on its own with the four ecological variables, and in combination with Amici et al.’s^75^ inhibition and detour indices (Table 7). While the inhibition index loads once again with group size and day journey length as in Table 6 and S8, their social inhibition task loads on the same factor as the detour task and diet (with home range being somewhat ambivalent in its loadings). This suggests that this task may not be indexing the same underlying phenomenon as the A-not-B and indifference tasks.

**Table 6:** Factor loadings (with varimax rotation) for the variables for each of the three datasets.

	Stevens^59^		MacLean et al.^58^				Amici et al.^75^		
	Indifference task		A-not-B task only		A-not-B and cylinder tasks		Inhibition† and Causality tasks‡		
Factors:	1	2	1	2	1	2	1	2	3
Inhibition task	**0.898**	0.249	**0.855**	0.053	**0.849**	0.189	**0.871**	0.308	-0.122
Cylinder task					0.593	**0.740**			
Causal reasoning/detour tasks							0.191	**0.955**	0.010
Group size	**0.885**	0.102	**0.900**	0.031	**0.888**	0.030	**0.915**	0.027	0.250
Day journey	**0.969**	-0.090	**0.895**	-0.180	**0.915**	-0.091	**0.915**	-0.237	-0.238
Diet (% fruit)	-0.018	**0.778**	0.020	**0.971**	-0.156	**0.931**	-0.036	0.102	**0.984**
Range size	0.148	**0.714**	0.498	0.208	0.485	0.356	-0.123	**0.941**	0.145
Species sampled	13		18		14		7		
Variance explained	74.9%		72.3%		75.7%		92.8%		

Extraction based on λ = 1.0. Bold font indicates variables that have a strong positive load (> 0.700) on the same factor.

† mean of two inhibition tasks (A-not-B and delayed gratification tasks).

‡ mean of three detour tasks (middle cup, plexiglass and swing door).

**Table 7:** Factor loadings (with varimax rotation) for the three cognitive variables from Amici et al.^75,76^.

	Social inhibition only		All three cognition tasks	
Factors:	1	2	1	2
Social inhibition task*	**0.880**	0.297	**0.855**	0.371
Inhibition tasks †			0.234	**0.922**
Causal reasoning tasks ‡			**0.970**	− 0.055
Group size	0.211	**0.919**	0.154	**0.899**
Day journey	− 0.213	**0.955**	− 0.268	**0.930**
Diet (% fruit)	**0.880**	− 0.237	**0.834**	0.080
Range size	**0.838**	− 0.010	-0.217	**0.893**
Species sampled	6		6	
Variance explained (%)	84.9		86.2	

Extraction based on λ = 1.0. Bold font indicates variables that have a strong positive load on the same factor.

* from Amici et al.^76^.

† mean of two inhibition tasks (A-not-B and delayed gratification tasks); from Amici et al.^75^.

‡ mean of three causal reasoning tasks (Middle cup, Plexiglass hole and Swing door); from Amici et al.^75^.

It was not possible to run a factor analysis of the Shultz & Dunbar^77^ executive function dataset because the number of species sampled varies between tasks. Instead, we ran separate analyses for the individual tasks. In two cases, the PCA yielded a single factor; since we are interested in whether the task being tested segregates with the social or ecological variables, we forced these into a two factor model. Table 8 summarises the results for the five most relevant tasks; Table S11 gives the results for the other four tasks. The Oddity, 3D-Oddity and Reversal (one-trial learning) tasks load strongly with the social factors, while the Detour and Reverse Reward tasks (essentially mapping tasks) load strongly with the ecological factors; in contrast, the String task (a pattern recognition task) loads with range size (but not diet), whereas the Displacement, Delayed Reward and Learning Set tasks (all essentially memory tasks) load ambivalently (but slightly favour the ecological subset). (Note that the Delayed Reward task in this set is not the same as the indifference task in the Stevens and Amici datasets.) There are sufficient data to include two tasks in the same analysis only for the Reversal Learning and Learning Set tasks: with a forced 2-factor solution, the first loads unambiguously with the social variables, the second with diet (Table S12). Note that on all these tasks, prosimians score lower than anthropoids, with the two callitrichid species sampled ranking with the prosimians rather than the anthropoids, as the Passingham-Wise Conjecture would predict (Fig. S3).

**Table 8:** Factor loadings (with varimax rotation) for the executive function tasks in the Shultz & Dunbar^77^ dataset.

	Oddity task		Detour task*		Reversal learning*		Delayed reward		Reversed reward*	
	(inference)		(mapping)		(one-trial learning)		(memory)		(rule inference)	
Factors:	1	2	1	2	1	2	1	2	1	2
Executive function task	**− 0.908**	− 0.117	0.612	**0.723**	**0.877**	-0.014	0.426	0.672	0.368	**0.729**
Group size	**0.982**	0.153	**0.899**	0.360	0.607	0.486	0.688	0.502	**0.962**	0.001
Day journey	**0.907**	-0.221	**0.969**	0.137	**0.907**	0.197	**0.929**	0.278	**0.799**	0.107
Diet (% fruit)	0.139	**0.923**	0.183	**0.966**	0.090	**0.938**	-0.791	0.160	-0.403	**0.681**
Range size	-0.313	**0.896**	0.857	0.514	**0.936**	0.281	-0.108	**0.900**	0.093	**0.870**
Species sampled	8		9		11		19		11	
Variance explained	89.4%		95.1%		81.6%		75.5%		72.7%	

Primary cognitive function is indicated in parenthesis below each task name.

Extraction based on λ = 1.0, except * where a 2-factor solution is forced. In both the latter cases, frugivory loaded weakly with the social variables and inhibition task (0.712 and 0.443, respectively, compared to 0.919 and 0.805 for the inhibition task).

Loadings for the other three tasks in this set are given in Table S11.

**Fig. 2:**
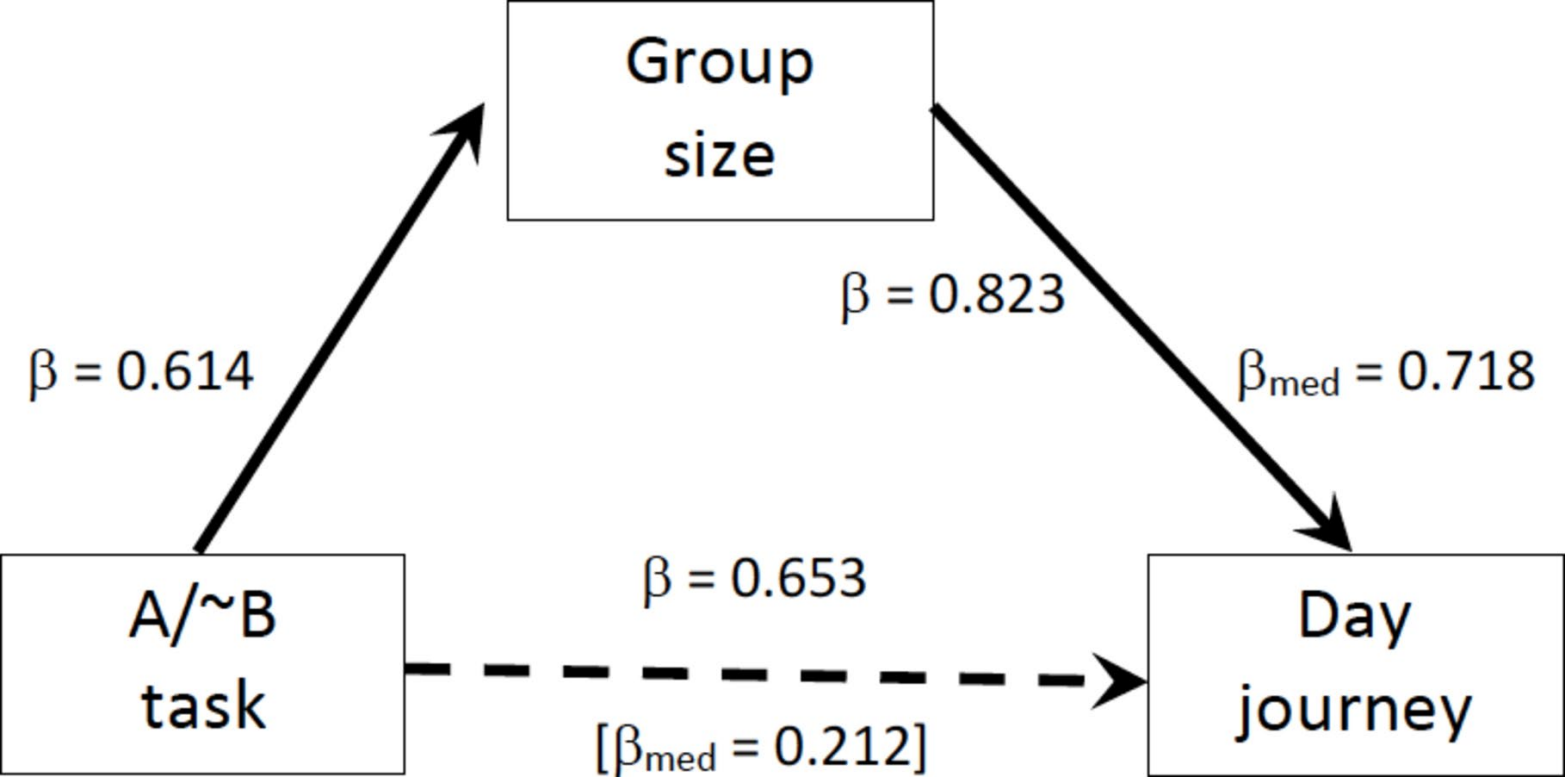
Mediation analysis of the relationship between the three variables in the social cluster for the A-not-B task in Table 4. A model with day journey length as the dependent variable gives a significantly better fit than one with group size as the dependent variable. βs are standardised slopes; β_med_ gives the standardised slope from the multiple regression equation with group size as the mediating variable.

To evaluate the relationship between self-control and its associated social variables in more detail, we ran a mediation analysis with the MacLean et al. A-not-B task (the inhibition index with the largest sample) as the independent variable and group size and day journey length alternately as dependent variable and mediator. A Sobel test indicates that a model with group size as mediator and day journey as dependent variable (Fig. 2) yields a significant model (z = 2.893, *p* = 0.0038). This model is significantly better than one with group size as the dependent variable and day journey as the mediator (z = 0.022, *p* = 0.982) or with inhibition as the dependent variable (z = 0.070, *p* = 0.944). This suggests that the capacity to inhibit behaviour (self-control) determines group size, and group size then determines day journey length.

### Passingham-Wise conjecture

**Fig. 3:**
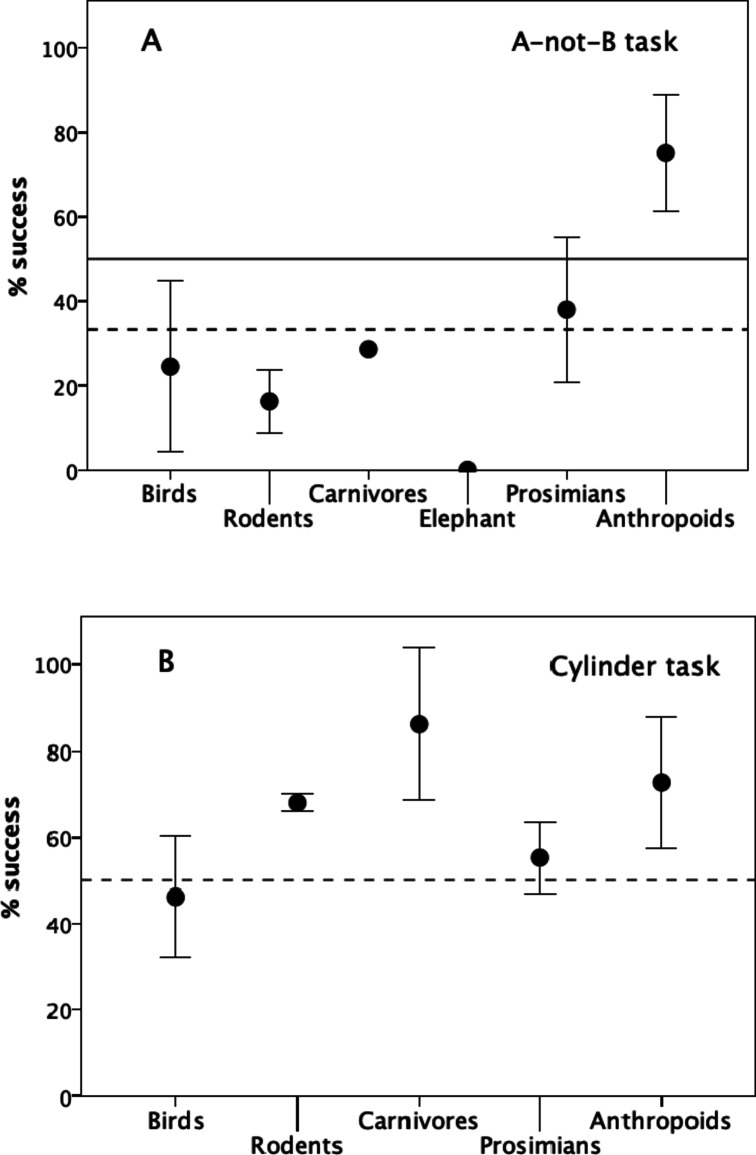
Performance on two cognitive tasks for different taxonomic groups for the two tasks in the MacLean et al. sample. (a) Mean (± 2se) percentage success on the A-not-B inhibition task. The dashed horizontal line denotes the chance response rate at 33% (for a task in which the animal chooses between three locations); the solid horizontal line indicates a chance response rate of 50% (assuming the animal only chooses between the two external cups and ignores the central cup). (b) Mean (± 2 se) percentage success on the cylinder task. The dashed horizontal line denotes the chance response rate at 50% (for a task in which the animal chooses between one of two locations). Number of species sampled for each taxon on the two tasks: birds (11,7 respectively), rodents (4,2), carnivores (3,2), elephant (1,1), prosimians (7,8), anthropoid primates (25,12). Data from^58^.

Finally, we use the two MacLean et al.^58^ tasks on the full range of mammal and bird species to test the Passingham-Wise Conjecture that inhibition of prepotent responses (self-control) only occurs in anthropoid primates. Figure 3a plots performance on the A-not-B task for the major taxonomic groupings. Performance varies significantly across the sampled taxa (F_6,19_=3.73, *p* = 0.013). Just as Passingham & Wise^57^ suggested, self-control is unique to anthropoid primates: they are the only taxon whose scores lie significantly above chance (irrespective of how this is defined) (for H_0_ = 33.3%: t_13_ = 8.09, *p* < 0.0001, 1-tailed positive). None of the non-anthropoid taxa perform at better than chance (for H_0_ = 33.3%: prosimian primates: t_5_ = 0.55, *p* = 0.312; rodents: t_0_ = 0.59, *p* > 0.330; birds: t_2_ = 0.77, *p* = 0.260). Indeed, MacLean et al.^58^ themselves confirm this: they report that the correlation between inhibition score and brain size is significant only in the anthropoids (phylogenetically controlled regressions: anthropoids: *p* < 0.01; non-anthropoid mammals: *p* = 0.71) (see also ^59,56^). Notice that, if the animals are making only a binary (correct/incorrect) decision (i.e. are ignoring the middle cup in this task), and hence H_0_ = 50.0%, then there is a strong suggestion for a perseveration effect in all the non-primates (they keep responding to the cup that was baited first, rather than the cup to which the reward was subsequently transferred). This suggests that non-anthropoids learn associatively after many failed trials, whereas anthropoid primates are able to unlearn a response very quickly (often after a single trial), just as Passingham & Wise^57^ suggested might be the case on the grounds that one-trial learning seemed to be a primate speciality.

Figure 3b plots the equivalent data for the cylinder task. In contrast to the A-not-B task, performance on the cylinder task does not differ significantly across the major taxonomic groups (F_5,25_=2.22, *p* = 0.084). If anything, carnivores might actually out-perform anthropoids on this task (albeit not significantly), with rodents running them a close second (1-tailed positive tests against H_0_ = 50% correct: anthropoid primates: t_10_ = 2.96, *p* = 0.007; prosimian primates: t_6_ = 1.25, *p* = 0.113; carnivores: t_(0)_ = 4.09, *p*≈0.075; rodents: t_(0)_ = 17.9, *p*≈0.330; birds: t_5_=-0.54, *p*≤0.806, with t_(0)_ tested as t_(1)_ for a maximal upper limit on the p-value). Once again, only anthropoid primates consistently perform with conviction on this task, though non-anthropoids generally do considerably better with this task than on the A-not-B task.

## Discussion

Our results allow us to draw three broad conclusions. First, the various tasks used to assay inhibition do not seem to index the same underlying cognition. Some tasks are better described as indexing mapping (how to get to some proximate goal), others as causal reasoning, rather than the capacity to inhibit prepotent responses so as to maximise long term opportunities. Second, using different cognitive tasks from four independent datasets, we showed that strategic inhibition (self-control) is correlated with key variables that affect group coordination (group size and day journey length), but not with indices that explicitly impact on food-finding decisions (percentage of fruit in the diet and home range size, with the latter being a proxy for decisions on where to find productive food patches). Third, we confirm the Passingham-Wise Conjecture: self-control defined as strategic inhibition (as reflected in A-not-B tasks or temporal discounting) appears to be unique to anthropoid primates, at least among the species currently available to test. This conclusion is given added support by the analyses of the Shultz-Dunbar executive function tasks: prosimians and callitrichids score lower than anthropoids on all the tasks except the detour task and the displacement task (both essentially mapping tasks). Neither prosimians nor callitrichids have a frontal pole^57^.

One important secondary finding is that the Amici et al. social inhibition task (a reversed reward task that both monkeys and apes find hard^96^; see also Fig. S1) is not a social inhibition task as such: it is only social in that it happens to involve a rival recipient rather than a wasted reward opportunity. It may best be described as indexing causal knowledge (see also^97^). These kinds of foraging-related competences (including delayed gratification, spatial displacement and the formation of learning sets as well as mapping and causal reasoning) may well be important in foraging contexts and hence widely distributed taxonomically. Cuttlefish, for example, perform well on both delayed gratification and reversed reward tasks^98^. De Petrillo et al.^99^ provide data on a number of executive function tasks in four lemur species that offer clear support for this. Although they claimed that these results support an ecological rather than a social basis for intelligence, we should be skeptical of this claim. First, of the three substantively relevant tasks (as opposed to those that index basic cognition), the four species perform at or near chance levels on two (A-not-B and reversal learning, both associated with sociality) and only two perform at consistently better than chance on the third (technically a delayed reward rather than a temporal discounting task, associated most closely with foraging). Second, the prosimians perform poorly compared to anthropoids (see also Fig. S3); this is correlated with the fact that they have significantly smaller brains and are socially less complex^56,77,93^. Third, as Fig. 3 demonstrates, only anthropoids are capable of strategic inhibition (self-control); since prosimians lack the socio-cognitive skills of anthropoids^56^, it would be surprising if their competencies reflected anything other than ecology. In fact, prosimians are about as social as carnivores, with the principal form being driven not by increasing group size but by pairbonded monogamy^56^. That prosimians might perform competently on some of these tasks does not explain the evolution of large social brains in anthropoid primates or the cognitive competences associated with these (a logical fallacy known as over-generalisation^70^).

That strategic inhibition is less widely distributed taxonomically (being limited to anthropoid primates) – and, within primates, is more unevenly distributed than other forms of cognition – probably reflects the fact that the skills involved are cognitively and neurophysiologically expensive, and hence covary with brain size^56,58,59,77^. We suugest that this is likely to be because the animal has to hold two versions of reality in mind at the same time: the world as it actually sees it and the world as it was in the past, or might be in the future. This depends critically on being able to prevent one model leaking into the other, and seems to require a high level of cognitive inhibition similar to that which makes mentalising more cognitively demanding than other forms of cognition in humans^52^.

Tasks that primarily involve causal reasoning (such as the cylinder task and other detour tasks) give more ambiguous results than the strategic inhibition tasks, and mainly load with diet. The lack of any clear difference in performance on these tasks between mammals and birds (Fig. 3b) reinforces the suggestion that these tasks index a generalised cognitive skill whose primary function is related to food-finding and other non-social forms of causality rather than strategic social decision-making. It is noteworthy that rodents and carnivores performed as well on the cylinder task as anthropoid primates, and did so significantly better than the other sampled orders. These three orders probably engage in far more manipulation of their food (or prediction of the future actions of prey, in the case of carnivores) than any of the other taxa.

Of particular interest in this context are the sciurids (squirrels): these have not, so far as we know, been tested directly on these kinds of task, but as a taxon characterised by considerable manual dexterity they would be especially interesting as a test of the hypothesis. Sciurids are of special interest in this context because the earliest Oligocene primates (the plesiadapids) were squirrel-like in diet, habitat and brain size (and organisation)^100^. The plesiadapids eventually lost out in ecological competition with the squirrels during the ensuing Eocene, and either went extinct or moved into a new niche where they evolved into modern primates^101^. It may be that their squirrel-like ancestry played an important role in providing a precursor brain that was capable of handling complex causal processes as distinct from simple manual dexterity.

Although several authors have claimed that birds^62–65^ perform just as well as apes and monkeys on the cylinder task, most of the species tested (mainly parrots and corvids) are ones with large brains (for birds), possess sophisticated cognitive abilities and regularly manipulate food so as to extract it from a matrix (as well as being highly social). In fact, Duque & Stephens^102^ note that non-corvids generally perform at or below chance level on this task (see also^63^). More importantly, perhaps, a recent lesion study of a large sample of macaques confirmed that reversal learning is especially dependent on the granular orbitofrontal cortex (OFC) including the frontal pole (BA 10/11/12) (as well as intact connections with the amygdala and hippocampus, and, in the temporal lobe, the rhinal cortex – the latter being associated with object recognition): animals with lesions in these regions took longer to switch behavioural response after the reward schedule reversed^103^. In humans, the OFC is known to be involved in mentalising^104^. In effect, lesioned animals could no longer do one-trial learning, and instead reverted to something closer to conventional association learning. These results add weight to the suggestion that strategic inhibition and causal reasoning may have evolved independently of each other, and thus represent a case of mosaic evolution in cognitive skills and their underlying neural bases (see also^30,105^).

Inevitably, our analyses are limited by the modest number of species for which data are available, as well as by the types of tasks that have been used in these kinds of experiments. Of particular importance is the fact that, aside from elephants, none of the nonprimate species studied have bonded social groups; it may be that tests on species that do (e.g. equids, delphinids, tylopods) reveal a capacity for passing self-control tasks. Studies of a wider range of species are obviously needed to test the Passingham-Wise Conjecture more thoroughly than we have been able to do.  The issue here hinges on whether any non-anthropoids have brain region BA10 (frontal pole) since this seems to be critical for being able to solve reversal tasks by one-trial learning in the way anthropoid primates do^57^. Although some effort has been made to design studies with more explicitly social outcomes^106–109^, the conception of social tasks in these cases bears little resemblance to those that actually drive these species social and ecological lives in the wild. Recently, however, more sophisticated experimental^110^ and longterm observational^15,17–19,21,22,111,112^ studies on wild populations have started to become available. Davidson et al.^113^, for example, designed an ecologically more appropriate version of the cylinder task and found very high success rates (~ 75%) in great tits (although the design lacks an obvious baseline for random performance, so their results are not directly comparable to those in Fig. 3b).

Although many of the tasks considered herein have been described as testing for inhibition, in fact they correlate poorly with each other (Table 4) (see also^67^). The cylinder task, by its very nature, is essentially a ‘detour task’^69^: it asks whether animals recognise that to obtain a food reward they have to do so via a side route at either open end of the tube rather than by directly reaching for it through the plastic tube. Consideration of the task demands involved suggests that it might, in fact, be better characterized as a causal reasoning task that only requires motor (action) inhibition. In this respect, the demands it places on the animal are similar to the puzzle box task used in a study of mental rehearsal: in this study, animals had to be able to decide which of several alternative routes into the box would allow them to access a clearly visible reward. Orang utans, chimpanzees and children all solved the problem faster after an opportunity to visually inspect (but not touch) the boxes (i.e. an opportunity to mentally rehearse possible solutions) than if they were presented with the boxes without prior inspection, with the success and speed with which they did so correlating with species’ frontal lobe volume^114^.

There are, of course, interesting questions to be asked about behaviours such as caching which occur widely both in mammals (e.g. squirrels^115^) and birds (scrub jays, titmice^116,117^), especially where there is a distinction between obligate caching and tactical caching in response to being watched^118^. Even in the latter case, however, we need to be careful to distinguish between responses based on associative learning and responses based on inference or one-trial learning in novel situations since these are very different processes and not equally distributed taxonomically^57^.

Mediation analysis makes it clear that by far the best model of the causal relationships between the three variables in the social cluster is that the capacity to inhibit prepotent actions determines group size, and group size then determines day journey length. Biologically, this makes more sense than any alternative model. In the absence of the capacity to maintain group coherence, foraging groups will fragment and disperse, resulting in a proportional loss in the benefits of grouping. This suggests that self-control plays a crucial role in managing group size and its constituent social relationships rather than influencing day journey length directly. Unfortunately, all psychological experiments inevitably use food as a reward (mainly because this simplifies experimental designs by allowing experiments to be kept short), and this seems to have had the misleading consequence that everyone inevitably assumes that food is the primary motivation for animals^58,59^, when this is not necessarily the case for intensely social species^30^. Greater caution may need to be exercised in the assumptions we make about what particular tests actually mean in terms of underlying cognitive skills and the functional objectives these are designed to achieve in animals’ lives.

These results feed into a longstanding distinction drawn between species that have large stable social groups (congregations) and those that live in unstable herds (aggregations, or fission-fusion social systems)^30,31^. The former are characterized by intense affiliative relationships between individuals, mediated in primates by social grooming^86,119,120^ and the constant monitoring of social partners^32^, as well as animals’ willingness to act altruistically towards each other^121^. Being able to distinguish between degrees of social relatedness, and make judgments about how to allocate time and effort strategically between different individuals is central to the temporal cohesion and coherence of social congregations^105^. The capacity to inhibit and modulate behaviour is also crucial in this context in order to ensure that individuals synchronise their movements so as not to lose contact with important social partners while foraging: when one stops to rest, the other must inhibit its desire to continue foraging and go to rest as well. Of the non-anthropoid species studied by MacLean et al.^58^, only elephants have bonded sociality above the level of monogamous pairbonds. That the seven Asian elephants in their sample failed comprehensively on the A-not-B task (Fig. 3a) is surprising, but similarly poor results have since been obtained with four African elephants^122^. (The data suggest that the one successful individual solved the reversal learning task slowly by conventional association learning, not instantaneously by one-trial learning^122^). However, elephants have a fission-fusion social system that does not depend on maintaining the cohesion of large social groups^123^, and this might possibly explain why they seem to score poorly on this task.

In conclusion, we draw attention to several important avenues that would merit future exploration. One, inevitably, is that tests on a wider range of non-primate species (especially those that live in stable, i.e. bonded, social groups) would clarify a number of uncertainties. A second consideration is that rather than treating subjects as isolated individuals, more attention needs to be given to running experiments on socially living animals with established relationships. A third is that more careful dissecting of the cognitive demands of the different tasks is needed. In this respect, fMRI neuroimaging of the brain’s activity during problem-solving are needed, although correlational studies of individuals’ performance with structural MRI of brain region size would provide useful insights. The principal message, however, is that much greater care needs to be taken in the inferences we make when using different tasks. Too often, the terms inhibition and self-control are attached to any off-the-shelf task with little attention to what the task actually involves. In many cases, this results in claims being made about the cognitive abilities of some taxa that are, realistically, neurologically implausible. A clearer understanding of the differences between motor, cognitive and behavioural inhibition than is currently evident in the literature will be necessary. When the inferences we make are based on a label and not on the task demands, we risk muddying the waters.

## Electronic supplementary material

Below is the link to the electronic supplementary material.

Supplementary Material 1

Supplementary Material 2

Supplementary Material 3

Supplementary Material 4

## Data Availability

All data reported in this paper are provided within the manuscript or as supplementary information files: DATASET-1: Inhibition tasks; DATASET-2: Executive function tasks; DATASET-3: Passingham-Wise Conjecture.
