# Supplementary material for: Self-control has a social role in primates, but not in other mammals or birds
Source: Sci Rep. 2025 May 21;15:17566. doi: 10.1038/s41598-025-99523-6 (PMC12092587; doi:10.1038/s41598-025-99523-6)
Supplement: Supplementary file 1 — Supplementary Material 1 [file 41598_2025_99523_MOESM1_ESM.pdf]

Self-Control Has a Social Role in Primates, But Not in Other Mammals or Birds

R.I.M. Dunbar and **Susanne Shultz**

Electronic Supplementary Material

Supplementary Data

Table S1. Executive function tasks collated by Shultz & Dunbar [1]

Test	Description	Trait tested
Reversed reward	S chooses between two rewards but is rewarded with the one it doesn't choose [i.e. to get the larger reward it must choose the smaller]	Rule learning (inference)
Delayed reward	Reward hidden under one of two cups; S chooses after a 30-sec delay	memory
Reversal learning	S rewarded on one of two stimuli until reaches criterion; then rewarded on the other stimuli	one-trial learning (inference)
Oddity problem	S is rewarded for choosing the odd one out on a 3-stimulus task [shapes in 2 dimensions]	classification/ inference
3D oddity problem	As above, but with physical objects	classification/inference
String	S chooses between multiple strings, only one of which is attached to the reward	pattern recognition (mapping)
Learning set	S has to generalise a learned concept to a new set of stimuli (concept transference)	memory
Displaced reward	S watches reward being placed under a cup; reward is moved to a second cup while occluded from S's view [differs from A-not-B task in that reward moves, but not the cups, and that the move itself is occluded behind a screen]	object permanence/ memory
Detour	S has to remove a reward threaded onto a bent wire	detour

Species sampled [number of tasks tested, out of 8]

Ateles geoffroyi	[3]	*Lagothrix lagotricha*	[3]	*Nycticebus coucang*	[1]
Callithrix jacchus	[2]	*Lemur catta*	[4]	*Pan paniscus*	[4]
Cebus albifrons	[2]	*Macaca arctoides*	[2]	*Pan troglodytes*	[8]
Cebus capuchinus	[2]	*Macaca fascicularis*	[3]	*Papio cynocephalus*	[2]
Cercocebus atys	[1]	*Macaca fuscata*	[2]	*Papio hamadryas*	[1]
Cercocebus torquatus	[3]	*Macaca mulatta*	[8]	*Papio ursinus*	[1]
Cercopithecus mona	[1]	*Macaca nemestrina*	[6]	*Pongo pygmaeus*	[6]
Cercopithecus nictitans	[3]	*Macaca nigra*	[2]	*Saguinus oedipus*	[2]
Chlorocebus aethiops	[1]	*Macaca silenus*	[2]	*Saimiri sciureus*	[6]
Eulemur fulvus	[1]	*Macaca sylvanus*	[1]	*Sapajus apella*	[3]
Eulemur macacao	[2]	*Mandrillus leucophaeus*	[1]	*Semnopithecus entellus*	[1]
Gorilla gorilla	[6]	*Mandrillus sphinx*	[2]	*Varecia variegata*	[2]
Hylobates lar	[2]	*Miopithecus talapoins*	[1]		

All of these tasks have a wide distribution of values across species (Table S2).

Table S2. Range in species' performance on the executive function tasks of Table S1.

Task	Mean % correct Minimum	Maximum
Reversed reward	18.7	49.0
Delayed reward	45.0	100.0
Reversal learning	38.0	86.0
Oddity problem	64.0	86.5
3D oddity problem	45.0	79.3
String	79.0	96.0
Learning set	50.8	94.0
Displaced reward	19.0	97.5
Detour	0	98.2

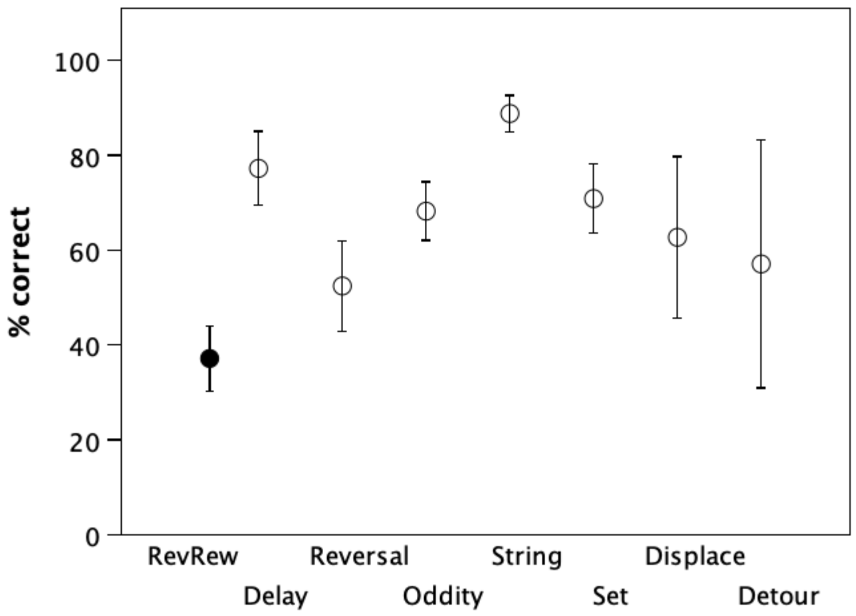

Fig. S1. Relative difficulty of the executive function tasks, indexed as the mean±95%CIs for percent correct trials by 39 primate species from 21 genera (see Table S1). RevRew = reversed reward task; Set = learning set. Sample sizes are (L to R): 11, 19, 11, 8, 12, 17, 9 and 9 species. Source [1]

Diet data

We sourced our data on diet from Powell et al. [2] rather than DeCasien et al. [3] because we considered the Powell data compilation more reliable. However, the value of 8.5% that they give for *Macaca mulatta* is based on just one idiosyncratic high altitude study site. Other more typical habitats give much higher values of frugivory for this species. We searched for diet data for this species on GoogleScholar and located a further five studies (Table S3). We use the mean of these studies in our analyses.

Table S3. Diet data for *Macaca mulatta*

Study site	Country	% fruit in diet	Source
Taihangshan Reserve	China	36.5	Cui et al. [4]
Nonggang Reserve	China	30.0	Tang et al. [5]
*Murree Hills	Pakistan	8.5	Goldstein & Richard [6]
Buxta Tiger Reserve	Bangladesh	74.9	Sengupta & Radhakrisha [7]
Asola-Bhatti Sanctuary	India	5.7	Ganguly & Singh Chauhan [8]
Siwalik Hills	India	63.0	Lindburg [9]
**Mean**		**37.2%**	

* Site on which Powell et al. [2] based their estimate.

Powell et al. [10] do not give a value for percent of fruit in diet for *Saguinus oedipus*. We use the value given for this species by Garber [11].

We did not use Powell et al. [2] as a source of data for day journey length or home range size because, although their values for day journey correlate significantly with those we compiled from Smuts et al. [12] and Campbell et al. [13] (r=0.887, p=0.003), those for range size, in particular, appear to be based on a very selective sampling of study sites. Our sources are based on a wider range of primary sources, and are likely to be more representative.

Fission, group size and day journey in *Papio* populations

Table S4. Fissioning index

Index	Definition
0	Group relatively compact during foraging, and always sleeps together; group spread during foraging always <200m
1	Group becomes dispersed during foraging (mean spread >200m), but always sleeps together
2	Group fragments during foraging, with sub-groups moving independently, with subgroups sometimes sleeping at separate sites

For each study site, index is based on descriptions of foraging patterns given by primary sources

Table S4 gives the data for individual baboon populations, and Table S5 gives the definitions for the fission index categories.

Table S5. Group size, day journey length and the fissioning index for a sample of baboon study sites

Site	Group size	Day journey (km)	Fissioning index†	Source
*Awash Station, Ethiopia	83.0	6.5	2	Nagel [14]
*ErerGota, Ethiopia	83.0	8.9	2	Sigg & Stolba [15]
*Awash Filoha, Ethiopia	190.0	7.5	2	Swedell [16]
Mt Assirik, Senegal	247.0	7.9	2	Byrne [17]; Sharman [18]
Siminti, Senegal	70.8	4.0	2	Zinner et al. [19]
Gashaka NP, Nigeria	28.4	2.4	0	Sommer & Ross [20]
Metahara, Ethiopia	87.0	5.8	1	Aldrich-Blake et al. [21]
Bole Valley, Ethiopia	19.0	1.2	0	R. Dunbar (unpublished)
Mulu, Ethiopia	22.0	1.1	0	R. Dunbar (unpublished)
Awash Falls, Ethiopia	71.0	5.3	1	Nagel [14]
Budongo Forest, Uganda	37.5	3.8	0	Paterson [22], pers. comm.
Chololo, Kenya ‡	102.0	5.6	1	Barton [23]
Gilgil, Kenya ‡	49.0	4.3	1	Harding [24]
Chololo STT 1986, Kenya	102.0	5.6	1	Barton [23]
Chololo PHG 1995, Kenya	25.0	4.6	0	Kenyatta [25]
Gombe NP, Tanzania	43.0	2.4	0	J. Oliver (pers. comm.)
Amboseli NP, Kenya [Hook]	46.5	6.1	1	D. Post (pers.comm.)
Ruaha NP, Tanzania	72.0	6.8	1	Collins [26]
Mikumi NP (1995), Tanzania	18.0	3.9	0	Hawkins [27]
Giants Castle, S. Africa	11.8	0.9	0	Henzi et al. [28]; R. Byrne (pers. comm.)
Cape Point, S. Africa	85.0	7.9	2	Davidge [29]
Honnet, S. Africa	77.0	9.0	2	Stoltz & Saayman [30]
Suikerbosrand, S. Africa	78.0	4.1	2	Anderson [31]
R. Kuiseb, Namibia	15.5	4.1	0	Brain [32]
Tsaobis, Namibia	34.3	5.5	0	King et al. [33]; G. Cowlishaw (pers. comm.)

* *Papio hamadryas*

† see Table S4

‡ based on a comparison of group size and the mean number of individuals within 10m of a focal adult

Exclusions and Anomalous Group Sizes

Daubentonia was omitted from the MacLean et al. [34] dataset because of doubts over the correct group size to use. Although *Daubentonia* (a very rare and difficult to study nocturnal prosimian) has been consistently listed with a group size of N=1 in most comparative datasets because it forages solitarily, in fact field studies have suggested that it actually lives in much larger communities (neighbourhoods) as do all the other nocturnal prosimians [35-37]. However, there are no reliable estimates of what this group size actually is (though values around N=8 have been suggested – i.e. considerably larger than the conventionally cited value of N=1).

Papio hamadryas was omitted from the MacLean et al. [34] dataset for related reasons. This species is unusual for a baboon in that it lives in a multilevel society with at least four different grouping levels [38]. It has never been clearly determined which of these grouping levels is the correct one to use in comparatrive studies. Although the band (mean size N=84.5) is commonly cited, there are good cognitive and socio-demographic grounds for considering the clan (mean size N=24.0) as the more appropriate natural grouping [39]. It is important to note that mean band size is 3.3 SDs above the overall mean for the MacLean [34] sample and the species' mean day journey length is 3.5 SDs above the sample mean. It is normally customary to exclude values that lie >2 SDs from the mean. For present purposes, we excluded the species, but give results for separate analyses with the two candidate group sizes in the ESM.

The orang utan (*Pongo*) provides another potentially problematic case: most comparative databases give a group size of N=1 for this species on the grounds that, like *Daubentonia*, it typically forages alone. However, these genus currently occupies a retreat habitat on the limits of its biogeographical range [40-41]. Subfossil orangs on the Chinese mainland occupied woodland rather than forest habitats and were likely much more terrestrial than they are now [42-43] and so almost certainly foraged in much larger groups. In fact, this species is more intensely social than the gorilla and is commonly kept in groups of up to 7 animals in captivity [45-45]. Indeed, some contemporary populations forage in larger groups [46-48]. Recent studies provide compelling evidence that communities of 12-15 are typical [49-50], and this value fits extremely closely with the size we would predict given its neocortex size and the ape social brain relationship [39,51]. As with *Daubentonia*, using the conventional group size of N=1 risks confounding foraging group size with social group size [52]. We use the value of N=14 given by [51].

Supplementary analyses

Bivariate correlations

Table S6. Bivariate Pearson correlations for the Amici [53] dataset

	MacLean. A-not-B	Amici A-not-B	Amici delay	Amici middle	Amici plexy	Amici swing door	Amici social
MacLean cylinder	0.722	0.538	0.558	0.289	**0.918***	0.473	0.540
MacLean A-not-B		**0.756***	0.321	0.177	0.578	0.165	0.037
Amici A-not-B			**0.734***	0.086	0.371	-0.088	0.169
Amici delayed reward				0.418	0.538	0.056	0.317
Amici middle cup					0.526	0.366	0.307
Amici plexy hole						0.454	-0.106
Amici swing door							0.537

Bold* values are significant at p=0.05 1-tailed for a positive correlation

Table S7. Bivariate Pearson correlations for the Stevens [54] and MacLean et al. [34] tasks

	Diet	Group size	Day journey	Home range
Indifference	r=0.206<br>p=0.544<br>N=11	r=0.676<br>p=0.022<br>N=11	r=0.866<br>p=0.001<br>N=11	r=0.258<br>p=0.444<br>N=11
A-not-B	r=-0.072<br>p=0.758<br>N=21	r=0.637<br>p=0.002<br>N=21	r=0.603<br>p=0.004<br>N=21	r=0.461<br>p=0.041<br>N=20
Cylinder	0.428<br>p=0.076<br>N=18	0.519<br>p=0.027<br>N=18	0.512<br>p=0.030<br>N=18	0.443<br>p=0.066<br>N=18
Diet		-0.086<br>p=0.695<br>N=23	-0.197<br>p=0.366<br>N=23	-0.06<br>p=0.769<br>N=23
Group size			0.813<br>p<0.001<br>N=24	0.365<br>p=0.087<br>N=23
Day journey				0.242<br>p=0.265<br>N=23

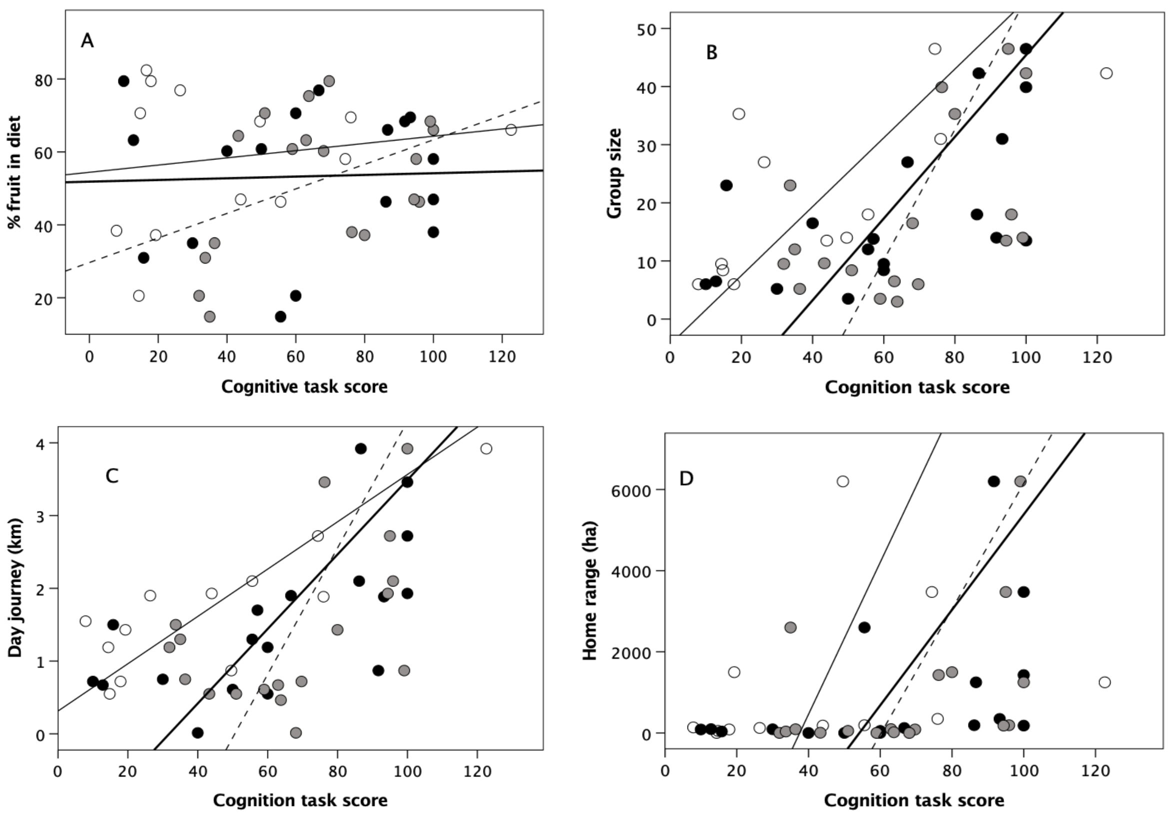

Figure S2. Cognition score as predictor of (a) diet (% fruit), (b) mean group size, (c) mean day journey length (km), and (d) mean home range area (ha) for individual species. Unfilled circles, thin solid line: MacLean et al. [34] A-not-B task; grey circles, dashed line: MacLean et al. [34] cylinder task; filled circles, thick line: Stevens [54] Go/No-go task. Lines are OLS regressions.

Table S8. PCA factor loadings (with varimax rotation) for the mean value of the social inhibition task from Amici et al. [55]

Factors:	1	2
Inhibition task† (mean)	**0.891**	0.048
Group size	**0.921**	0.128
Day journey	**0.885**	-0.403
Diet (% fruit)	-0.024	**0.780**
Range size	-0.004	**0.777**
Species sampled	7	
Variance explained (%)	76.4	

Extraction based on λ = 1.0. Bold font indicates variables that have a strong positive load on the same factor.
† Average of A-not-B and delayed gratification tasks

Table S9a. PCA factor loadings (with varimax rotation) for the variables for each of the three datasets, with orang utan (*Pongo*) excluded.

	Stevens [54] Indifference task		MacLean et al. [34] A-not-B task only		Amici et al. [53] Inhibition task ‡	
Factors:	1	2	1	2	1	2
Inhibition task	**0.889**	0.318	**0.831**	0.009	**0.864**	**-**0.156
Group size	**0.934**	-0.165	**0.933**	0.106	**0.933**	0.322
Day journey	**0.935**	0.150	**0.923**	-0.022	**0.859**	-0.257
Diet (% fruit)	0.054	**0.928**	0.035	**0.967**	0.067	**0.982**
Range size	**0.748**	-0.445	0.698	-0.415	**0.797**	-0.014
Species sampled	12		17		6	
Variance explained	86.2%		80.5%		83.1%	

Extraction based on $\lambda = 1.0$. Bold font indicates variables that have a strong positive load (>0.700) on the same factor.

‡ mean of two inhibition tasks (A-not-B and delayed gratification tasks)

Table S9b. PCA factor loadings (with varimax rotation) for the variables for the MacLean et al. [34] dataset, with *Papio hamadryas* included at two different grouping levels.

	MacLean [34] A-not-B task			
Grouping level:	Band (N=84.5)		Clan (N=24.0)	
Factors:	1	2	1	2
Inhibition task	0.568	0.535	**0.830**	0.077
Group size	**0.928**	0.059	**0.852**	0.050
Day journey	**0.947**	-0.056	**0.735**	-0.310
Diet (% fruit)	-0.275	**0.728**	0.040	**0.969**
Range size	0.414	0.591	0.603	0.075
Species sampled	22		22	
Variance explained	70.0%		67.4%	

Extraction based on $\lambda = 1.0$. Bold font indicates variables that have a strong positive load (>0.700) on the same factor.

Genus level analysis

Although there is negligible phylogenetic signal in any of the data and analyses of the data with and without phylogenetic control yield identical results [34,54], we nonetheless further checked whether phylogenetic inertia might distort our results by re-analysing the data as genus-level averages. Genus-level analysis removes most of the potential for phylogenetic inertia. In fact, there are only three genera with more than a single species sampled in either of the two datasets. The results are given in Table S9. As before, a factor analysis with λ=1 explains 70-74% of the variance, with the Go/No-Go and A-not-B tasks loading with group size and day journey length as before, and the cylinder task loading with diet and home range size. The only difference is that range size loads more strongly with diet on factor 2 in both datasets than was the case with the larger sample.

Table S10. PCA factor loadings (with varimax rotation and λ>1) for the five variables for each of the two datasets for mean genus-level data. Bold font indicates variables that load together on the same factor.

	Indifference task		A-not-B task		Cylinder task	
Factors:	1	2	1	2	1	2
Cognitive task	**0.774**	0.583	**0.853**	0.236	0.225	**0.902**
Diet	-0.252	**0.893**	-0.224	**0.801**	-0.648	**0.666**
Group size	**0.865**	-0.170	**0.783**	-0.333	**0.880**	0.216
Day journey	**0.881**	0.106	**0.853**	-0.188	**0.805**	0.277
Range size	0.200	**0.562**	**0.517**	**0.543**	0.231	**0.581**
Variance explained	74.4%		70.5%		73.3%	

Table S11. PCA factor loadings (with varimax rotation) for the four additional executive function tasks (see Table 8)

	Displacement (memory)		Learning set (memory)		String task (mapping)		3-D Oddity (inference)	
Factors:	1	2	1	2	1	2	1	2
Executive task	0.727	0.532	0.570	0.721	-0.315	**0.721**	**0.856**	0.429
Group size	-0.130	**0.753**	**0.921**	0.224	**0.829**	-0.356	**0.853**	0.221
Day journey	0.205	**0.895**	**0.956**	0.007	**0.942**	-0.250	**0.970**	-0.128
Diet (% fruit)	**0.951**	0.160	0.062	**0.784**	-0.817	-0.151	0.125	**0.992**
Range size	**0.844**	-0.288	0.062	**0.834**	0.061	**0.857**	0.862	0.505
Species sampled	9		17		12		7	
Variance explained	80.1%		79.8%		76.2%		93.0%	

Extraction based on λ = 1.0

Table S12. PCA factor loadings (with varimax rotation) for Reversal Learning and Learning Set tasks combined

Factors:	1	2
Reversal learning	**0.879**	0.361
Learning set task	0.461	**0.770**
Group size	**0.710**	0.497
Day journey	**0.981**	0.126
Diet (% fruit)	0.176	**0.959**
Range size	**0.885**	0.433
Species sampled	8	
Variance explained	89.3%	

Extraction based on λ = 1.0, with PCA forced to 2-factor solution

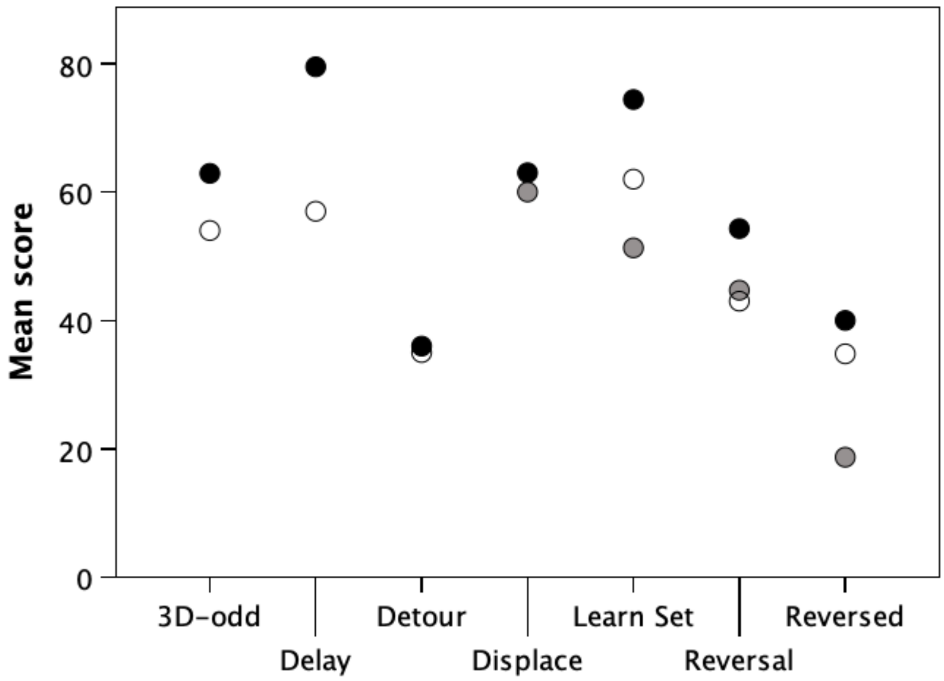

Fig. S3. Mean scores for Prosimians (unfilled symbols; 7 species), callitrichids (grey symbols; 2 species) and Anthropoids (filled symbols; 30 species) on seven of the executive function tasks from Table S1 for which there are data from two or more of these taxa.

References

[1] Shultz, S. & Dunbar, R.I.M. (2010). Species differences in executive function correlate with hippocampus volume and neocortex ratio across non-human primates. *Journal of Comparative Psychology* 124: 252-260.

[2] Powell, L.E., Isler, K. & Barton, R.A. (2017). Re-evaluating the link between brain size and behavioural ecology in primates. *Proceedings of the Royal Society, London*, 284B: 20171765.

[3] DeCasien, A.R., Williams, S.A. & Higham, J.P. (2017). Primate brain size is predicted by diet but not sociality. *Nature Ecology and Evolution* 1: 0112.

[4] Cui, Z., Shao, Q., Grueter, C. C., Wang, Z., Lu, J. & Raubenheimer, D. (2019). Dietary diversity of an ecological and macronutritional generalist primate in a harsh high-latitude habitat, the Taihangshan macaque (*Macaca mulatta tcheliensis*). *American Journal of Primatology* 81: e22965.

[5] Tang, C., Huang, L., Huang, Z., Krzton, A., Lu, C. & Zhou, Q. (2016). Forest seasonality shapes diet of limestone-living rhesus macaques at Nonggang, China. *Primates* 57: 83-92.

[6] Goldstein, S. J. & Richard, A. F. (1989). Ecology of rhesus macaques (*Macaca mulatta*) in northwest Pakistan. *International Journal of Primatology* 10: 531-567.

[7] Sengupta, A. & Radhakrishna, S. (2015). Fruit trait preference in rhesus macaques (*Macaca mulatta*) and its implications for seed dispersal. *International Journal of Primatology* 36: 999-1013.

[8] Ganguly, I. & Chauhan, N. S. (2018). Dietary preference and feeding patterns of the urban rhesus macaque *Macaca mulatta* (Mammalia: Primates: Cercopithecidae) in Asola-Bhatti Wildlife Sanctuary in India. *Journal of Threatened Taxa* 10: 12907-12915.

[9] Lindburg, D. G. (1977). Feeding behaviour and diet of rhesus monkeys (*Macaca mulatta*) in a Siwalik forest in North India. In: T.H. Clutton-Brock (ed) *Primate Ecology*, pp. 223-250. London: Academic Press.

[10] Powell, L.E., Isler, K. & Barton, R.A. (2017). Re-evaluating the link between brain size and behavioural ecology in primates. *Proceedings of the Royal Society, London*, 284B: 20171765.

[11] Garber, P. A. (1984). Proposed nutritional importance of plant exudates in the diet of the Panamanian tamarin, *Saguinus oedipus geoffroyi*. *International Journal of Primatology* 5: 1-15.

[12] Smuts, B.B., Cheney, D.L., Seyfarth, R.M., Struhsaker, T.T. & Wrangham, R.W. (eds.). (1987). *Primate Societies*. Chicago: University of Chicago Press.

[13] Campbell, C.J., Fuentes, A., Mackinnon, K.C., Bearder, S. & Stumpf, R. (Eds.) (2008). *Primates in Perspective*. Oxford: Oxford University Press.

[14] Nagel, U. (1973). A comparison of anubis baboons, hamadryas baboons and their hybrids at a species border in Ethiopia. *Folia Primatologica* 19: 104-165.

[15] Sigg, H., & Stolba, A. (1981). Home range and daily march in a hamadryas baboon troop. *Folia Primatologica* 36: 40-75.

[16] Swedell, L. (2002). Ranging behavior, group size and behavioral flexibility in Ethiopian hamadryas baboons (*Papio hamadryas hamadryas*). *Folia Primatologica* 73: 95-103.

[17] Byrne, R. W. (1981). Distance vocalisations of Guinea baboons (Papio papio) in Senegal: an analysis of function. *Behaviour* 78: 283-312.

[18] Sharman, M. J. (1982). *Feeding, ranging and social organisation of the Guinea baboon.* PhD thesis*,* University of St Andrews.

[19] Zinner, D., Klapproth, M., Schell, A., Ohrndorf, L., Chala, D., Ganzhorn, J. U., & Fischer, J. (2021). Comparative ecology of Guinea baboons (*Papio papio*). *Primate Biology* 8: 19-35.

[20] Sommer, V. & Ross, C. (Eds.). (2010). *Primates of Gashaka: Socioecology and Conservation in Nigeria's Biodiversity Hotspot* (Vol. 35). Berlin: Springer.

[21] Aldrich-Blake, F. P. G., Bunn, T. K., Dunbar, R. I. M., & Headley, P. M. (1971). Observations on baboons, *Papio anubis*, in an arid region in Ethiopia. *Folia Primatologica* 15: 1-35.

[22] Paterson, J.D. (1976). *Variations in Ecology and Adaptation of Ugandan Baboons Papio cynocephalus anubis*. PhD thesis, University of Calgary.

[23] Barton, R. A. (1990). *Foraging Strategies, Diet and Competition in Olive Baboons.* PhD thesis, University of St Andrews.

[24] Harding, R. S. (1976). Ranging patterns of a troop of baboons (Papio anubis) in Kenya. *Folia Primatologica* 25: 143-185.

[25] Kenyatta, C.G. (1995). *Ecological and Social Constraints on Maternal Investment Strategies*. PhD thesis, University College London.

[26] Collins, D. A. (1984). Spatial pattern in a troop of yellow baboons (Papio cynocephalus) in Tanzania. *Animal Behaviour* 32: 536-553.

[27] Hawkins, D.M. (1999). *Individual Time Budgets of Yellow Baboons in Mikumi National Park, Tanzania: Group Size and Environment.* PhD thesis, University of Liverpool.

[28] Henzi, S. P., Byrne, R. W. & Whiten, A. (1992). Patterns of movement by baboons in the Drakensberg mountains: primary responses to the environment. *International Journal of Primatology* 13: 601-629.

[29] Davidge, C. (1978). Ecology of baboons (*Papio ursinus*) at Cape Point. *African Zoology* 13: 329-350.

[30] Stoltz, LP & Saayman, G. (1970). Ecology and behaviour of baboons in the northern Transvaal. *Annals of the Transvaal Museum* 26: 99-143.

[31] Anderson, C. M. (1981). Subtrooping in a chacma baboon (*Papio ursinus*) population. *Primates* 22: 445-458.

[32] Brain, C. (1990). Spatial usage of a desert environment by baboons (*Papio ursinus*). *Journal of Arid Environments* 18: 67-73.

[33] King, A. J., Douglas, C. M., Huchard, E., Isaac, N. J. & Cowlishaw, G. (2008). Dominance and affiliation mediate despotism in a social primate. *Current Biology* 18: 1833-1838.

[34] MacLean, E.L., Hare, B., Nunn, C.L., Addessi, E., Amici, F., Anderson, R.C., et al. (2014). The evolution of self-control. *Proceedings of the National Academy of Sciences*, USA, 111: E2140-E2148.

[35] Iwano, T. (1991). An ecological and behavioral study of the aye-aye *(Daubentonia madagascariensis*). *African Studies Monographs* 12: 19-42.

[36] Ancrenaz, M., Lackman-Ancrenaz, I. & Mundy, N. (1994). Field observations of aye-ayes (*Daubentonia madagascariensis*) in Madagascar. *Folia Primatologica* 62: 22-36.

[37] Sterling, E. J. & McCreless, E. E. (2006). Adaptations in the aye-aye: a review. In *Lemurs: Ecology and Adaptation* (ed. L. Gould & M. L. Sauther), pp. 159-184. Berlin: Springer.

[38] Hill, R.A., Bentley, A. & Dunbar, R.I.M. (2008). Network scaling reveals consistent fractal pattern in hierarchical mammalian societies. *Biology Letters* 4: 748-751.

[39] Dunbar, R.I.M. & Shultz, S. (2021). Social complexity and the fractal structure of social groups in primate social evolution. *Biological Reviews* 96: 1889-1906.

[40] Dunbar, R.I.M. (1988). *Primate Social Systems*. Ithaca NY: Cornell University Press.

[41] Carne, C., Semple, S. & Lehmann, J. (2012). The effects of climate change on orangutans: a time budget model. In: L.M. Druyan (ed.) *Climate Models*, pp. 313-336. Rijeka (Croatia): InTech.

[42] Smith, R. J., & Pilbeam, D. R. (1980). Evolution of the orang-utan. *Nature*, *284*(5755), 447-448.

[43] Harrison, T.E., Xueping, J. & Su, D. (2002). On the systematic status of the late Neogene hominoids from Yunnan Province, China. *Journal of Human Evolution* 43: 207-227.

[44] Edwards, S. D., & Snowdon, C. T. (1980). Social behavior of captive, group-living orangutans. *International Journal of Primatology* 1: 39-62.

[45] Lardeux-Gilloux, I. A. (1997). *Social intelligence and dynamics in group-living orang-utans, Pongo pygmaeus pygmaeus.* PhD thesis*,* University of London.

[46] Galdikas, B. M. (1985). Orangutan sociality at Tanjung Puting. *American Journal of Primatology* 9: 101-119.

[47] Delgado Jr, R. A. & van Schaik, C. P. (2000). The behavioral ecology and conservation of the orangutan (Pongo pygmaeus): A tale of two islands. *Evolutionary Anthropology*: 201-218.

[48] Setia, T. M., Delgado, R. A., Atmoko, S. U., Singleton, I., & van Schaik, C. P. (2009). Social organization and male-female relationships. *Orangutans: Geographic Variation in*

[49] Mackinnon, J. (1974). The behaviour and ecology of wild orang-utans (*Pongo pygmaeus*). *Animal Behaviour* 22: 3-74.

[50] Singleton, I. & van Schaik, C.P. (2001). Orangutan home range size and its determinants in a Sumatran swamp forest. *International Journal of Primatology* 22: 877-911.

[51] Dunbar, R.I.M., MacCarron, P. & Shultz, S. (2018). Primate social group sizes exhibit a regular scaling pattern with natural attractors. *Biological Letters* 14: 20170490.

[52] Dunbar, R.I.M. & Shultz, S. (2023). Four errors and a fallacy: pitfalls for the unwary in comparative brain analyses. *Biology Reviews* 98: 1278-1309.

[53] Amici, F., Aureli, F. & Call, J. (2008). Fission-fusion dynamics, behavioral flexibility, and inhibitory control in primates. *Current Biology* 18: 1415-1419.

[54] Stevens, J.R. (2014). Evolutionary pressures on primate intertemporal choice. *Proceedings of the Royal Society, London,* 281B: 20140499.

[55] Amici, F., Call, J., Watzek, J., Brosnan, S. & Aureli, F. (2018). Social inhibition and behavioural flexibility when the context changes: a comparison across six primate species. *Scientific Reports* 8: 1-9.

[56]
